# Evaluation and Management of Respiratory Illness in Children With Cerebral Palsy

**DOI:** 10.3389/fped.2020.00333

**Published:** 2020-06-24

**Authors:** Rachael Marpole, A. Marie Blackmore, Noula Gibson, Monica S. Cooper, Katherine Langdon, Andrew C. Wilson

**Affiliations:** ^1^Department of Respiratory and Sleep Medicine, Perth Children's Hospital, Perth, WA, Australia; ^2^Research, Ability Centre, Perth, WA, Australia; ^3^Telethon Kids Institute, Perth, WA, Australia; ^4^Department of Physiotherapy, Perth Children's Hospital, Perth, WA, Australia; ^5^Department of Neurodevelopment and Disability, Royal Children's Hospital, Melbourne, VIC, Australia; ^6^Developmental Disability and Rehabilitation Research, Murdoch Children's Research Institute, Melbourne, VIC, Australia; ^7^Department of Paediatric Rehabilitation, Perth Children's Hospital, Perth, WA, Australia; ^8^Department of Paediatrics, The University of Western Australia, Perth, WA, Australia

**Keywords:** cerebral palsy, respiratory, evaluation, management, health services

## Abstract

Cerebral palsy (CP) is the most common cause of disability in childhood. Respiratory illness is the most common cause of mortality, morbidity, and poor quality of life in the most severely affected children. Respiratory illness is caused by multiple and combined factors. This review describes these factors and discusses assessments and treatments. Oropharyngeal dysphagia causes pulmonary aspiration of food, drink, and saliva. Speech pathology assessments evaluate safety and adequacy of nutritional intake. Management is holistic and may include dental care, and interventions to improve nutritional intake, and ease, and efficiency of feeding. Behavioral, medical, and surgical approaches to drooling aim to reduce salivary aspiration. Gastrointestinal dysfunction, leading to aspiration from reflux, should be assessed objectively, and may be managed by lifestyle changes, medications, or surgical interventions. The motor disorder that defines cerebral palsy may impair fitness, breathing mechanics, effective coughing, and cause scoliosis in individuals with severe impairments; therefore, interventions should maximize physical, musculoskeletal functions. Airway clearance techniques help to clear secretions. Upper airway obstruction may be treated with medications and/or surgery. Malnutrition leads to poor general health and susceptibility to infection, and improved nutritional intake may improve not only respiratory health but also constipation, gastroesophageal reflux, and participation in activities. There is some evidence that children with CP carry pathogenic bacteria. Prophylactic antibiotics may be considered for children with recurrent exacerbations. Uncontrolled seizures place children with CP at risk of respiratory illness by increasing their risk of salivary aspiration; therefore optimal control of epilepsy may reduce respiratory illness. Respiratory illnesses in children with CP are sometimes diagnosed as asthma; a short trial of asthma medications may be considered, but should be discontinued if ineffective. Overall, management of respiratory illness in children with CP is complex and needs well-coordinated multidisciplinary teams who communicate clearly with families. Regular immunizations, including annual influenza vaccination, should be encouraged, as well as good oral hygiene. Treatments should aim to improve quality of life for children and families and reduce burden of care for carers.

## What Is Cerebral Palsy?

Cerebral palsy (CP) is a heterogeneous group of disorders, caused by a non-progressive lesion in the developing brain. Most children have a normal musculoskeletal system at birth, but develop problems with posture over time (1). There are often associated disturbances in sensation, perception, cognition, communication, and behavior (2).

Prevalence of CP is 1.4 per 1,000 live births (3). Hemiplegia and diplegia are the most common topographical distributions of motor impairment (4). The Gross Motor Functional Classification System (GMFCS) describes gross motor functioning in CP, see Table 1 (5). GMFCS V describes the most severely impaired individuals, with inability to maintain antigravity head and trunk position or self-mobilize.

**Table 1:** Gross motor functional classification system.

**Level**	**Description**
I	Walks without restrictions. Limited in advanced gross motor skills.
II	Walks without aids, limited mobility in the community and outdoors.
III	Walks with aids. Uses wheelchair for long distances.
IV	Self mobility with powered mobility
V	Severely limited, unable to lift head and trunk or use powered mobility due to other comorbidities like vision impairment.

## Why Is Respiratory Illness in Cerebral Palsy Important?

The commonest cause of morbidity and mortality in people with CP is respiratory-related (6). Early mortality is more common in people with severe or profound intellectual disability, severe motor impairment, epilepsy, spasticity, and term birth (7). Compared to the general population, adults with CP have a 14-fold risk of death from diseases of the respiratory system (8). Although individuals with CP are surviving longer than previously (6, 9), respiratory failure has been the leading cause of death in this population since the 1970's (10, 11).

More severely impaired children with CP are admitted to hospital more often and for longer (12, 13), have more procedures and diagnoses per admission, and are more likely to die while in hospital.

Hospitalization and pain are the two strongest predictors of poor health-related quality of life (QoL) in children with severe CP (14). As the commonest cause of medical admission to hospital in severe CP is respiratory illness, it follows that respiratory illness is a leading cause of reduced health-related QoL. There is also a significant increase in the frequency of hospitalizations in children with severe physical disability in the last 6 months of life (15). Therefore, referral to palliative care should be considered if the frequency of admissions increases.

## Risk Factors for Respiratory Illness

**Figure 1:**
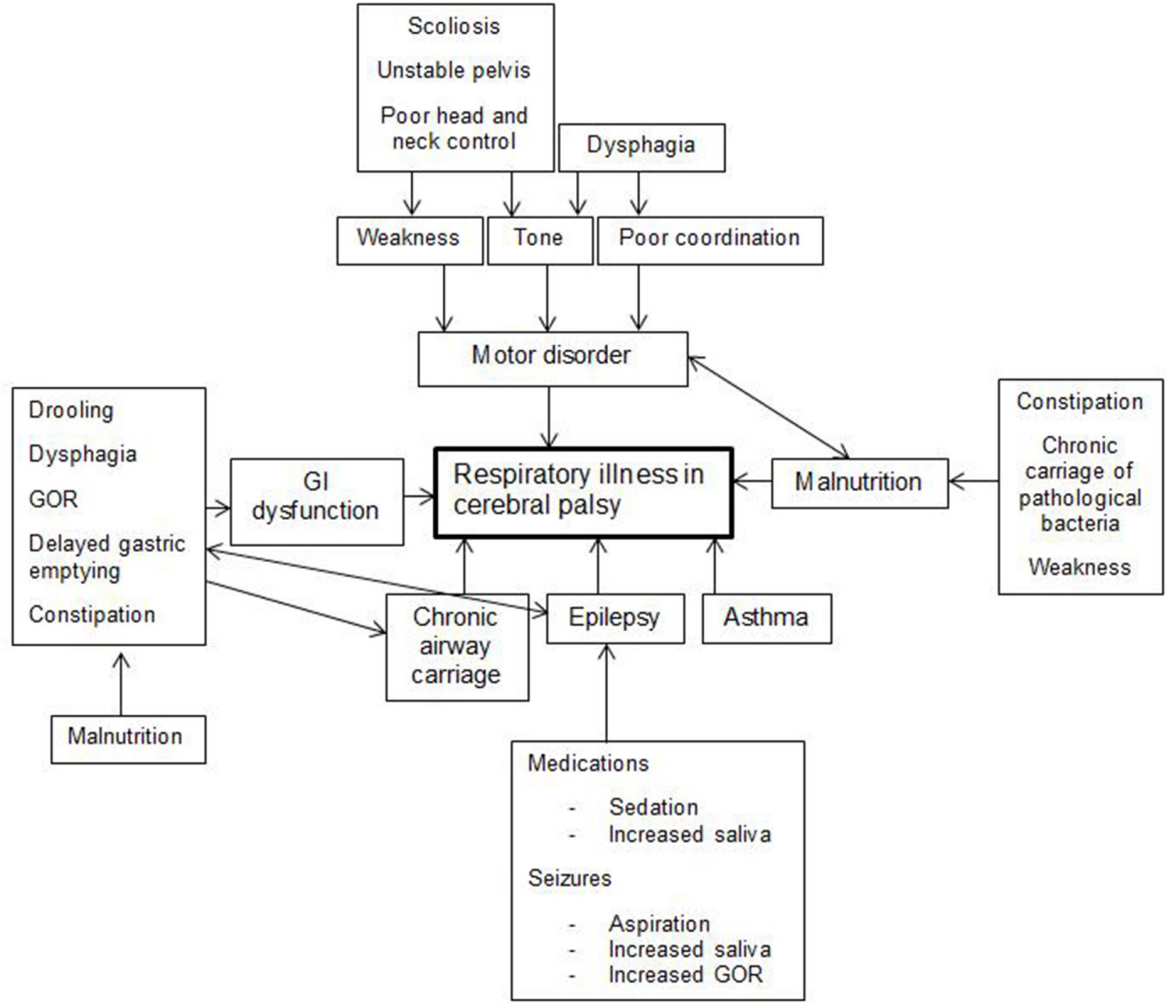
Multiple factors play a role in respiratory illness in cerebral palsy. GI, Gastrointestinal; GOR, Gastroesophageal reflux.

Respiratory illness in CP is a complex multifactorial disease process that is not well-understood. It can present with recurrent episodes of respiratory failure, mild illnesses, and/or chronic slowly progressive disease. It is probably caused by a combination of a chronic suppurative process and respiratory impairment similar to neuromuscular disease. The main driver of this process is aspiration of saliva, food, liquid, and refluxate with inability to evacuate secretions effectively. The motor disorder, malnutrition, and chronic carriage of pathogenic bacteria also contribute (16, 17). So do prematurity and bronchopulmonary dysplasia in some children. Factors contributing to respiratory disease are summarized in Figure 1.

The prevalence of respiratory symptoms in young people with CP is high (18). In a cross-sectional survey of 551 participants aged 1–26 years old, 46% reported a gurgly voice, wheezing, coughing, sneezing, choking, vomiting, or regurgitating during, or after meals. Individuals classified as GMFCS IV and V had more symptoms. Participants who took nil by mouth also had mealtime symptoms and even more hospital admissions than oral eaters (18, 19). This is probably owing to oropharyngeal dysphagia affecting saliva management, and/or gastro-esophageal reflux (GER) with secondary aspiration, or to an established, pre-existing disease continuing despite mealtime interventions.

A prospective data-linkage study with young people with CP identified factors significantly associated with subsequent respiratory hospitalizations over a 5-years period. They included: GMFCS Level V, at least one respiratory hospital admission in the previous year, at least two courses of antibiotics in the previous year, oropharyngeal dysphagia, frequent respiratory symptoms (daily cough, or weekly sounding chesty, phlegmy, or wheezy), current seizures, GER, mealtime symptoms when well (gurgly voice, wheezing, coughing, sneezing, choking), and nightly snoring (20, 21).

In these studies, both dysphagia and gross motor function level were strongly associated with respiratory illness. However, dysphagia was the more important factor: children with severe gross motor function levels without dysphagia did not have an elevated risk of respiratory illness; whereas children with dysphagia had an elevated risk of respiratory illness, regardless of their gross motor function level (19).

Although risk factors for respiratory illness in CP are known, there is weak evidence to date regarding the respiratory consequences when these risk factors are modified (22). Nevertheless, there is broad agreement about the mechanisms by which those risk factors predispose to respiratory illness and about the appropriate clinical pathways to be adopted [Myers et al. provide a recent overview (23)]. The present review will discuss each risk factor, its association with respiratory illness, the management options to be considered, and their advantages and disadvantages.

### Oropharyngeal Dysphagia: Aspiration of Food and Drink

Since aspiration is a risk factor, its treatment would be expected to reduce respiratory illness. Aspiration can arise from oropharyngeal dysphagia (food, drink, medication, and saliva) or from GER (stomach acid and contents).

Oropharyngeal dysphagia is common in young people with CP but is generally silent (24). It is caused by incoordination of swallowing with breathing, caused by the neurological lesion impacting tone and strength of the muscles of the jaw, cheeks, lips, tongue, palate, and pharynx, and by sensory impairments. Epilepsy and medication side-effects causing sedation also affect children's ability to swallow safely. Dysphagia is more prevalent in younger children and those classified as GMFCS III-V [who are also less likely to improve over time (25, 26)]. Dysphagia is associated with lower body weight, lower BMI, and parental stress (27). At 18–36 months, 39% of children with CP have modified food textures and around 10% are enteral tube fed. Children with higher GMFCS levels are more likely to receive the majority of their energy requirements from fluids, yet fluids pose the highest risks of aspiration (28).

Assessment of dysphagia by a speech therapist is necessary to ensure safety of oral feeding and its adequacy to meet nutritional needs. Assessment may include pulse oximetry, cervical auscultation, and videofluoroscopy swallow study (VFSS). The clinical signs of pharyngeal phase impairment include wet voice, wet breathing, and cough. These are good clinical markers of aspiration on thin fluids when compared with VFSS results (29).

Other assessments/classifications include the Eating and Drinking Ability Classification System (EDACS), and Dysphagia Disorders Survey**—**Pediatric Part 2.

The EDACS is for children with CP aged 3 years and above. EDACS Level I describes safe and efficient swallowing. At Level V, the child cannot eat and drink safely, relying on tube feeding. The EDACS strongly correlates with the GMFCS (30).

The Dysphagia Disorders Survey Part 2 assesses functional eating competency. It screens for oral, pharyngeal and esophageal dysphagia in individuals with developmental disability (31). Where ongoing concerns exist, a VFSS and flexible endoscopic evaluation of swallow can be done. A VFSS reviews the oral and pharyngeal phases of swallowing under direct vision. A flexible endoscopic evaluation of swallow allows visualization of the vocal cords and parts of the pharyngeal phase. Both describe a brief window in time and the agreement between them is low (24). Changes in chest Computed Tomography scans are seen in children aspirating saliva on flexible endoscopic evaluation of swallow, including bronchial wall thickening and atelectasis. The majority of these children had CP (32). While both dysphagia and aspiration lung disease can be objectively demonstrated, it can be a difficult conversation when a child is silently aspirating and is unsafe but has not yet had any acute respiratory illnesses. If other complications of dysphagia are present, like failure to thrive, prolonged feeding and inability to trust others to feed the child, then these may provide an opportunity for conversation about other feeding options.

Management of dysphagia is holistic and includes regular, thorough oral care with regular dentist visits, non-stressful feeding experiences, and nutritional maintenance of overall health (24). Food modifications can include changes to textures to reduce aspiration (33, 34). Oral sensorimotor therapy does not produce weight gain or improve feeding efficiency (35). Feeding should be done in the appropriate position (with postural trunk and head support), at the right time (avoiding fatigue and postictal states), in the right environment, with appropriate utensils, and by the right person (33). If oral feeding is unsuccessful under these conditions, tube feeding needs to be considered with the family. If non-orally fed, most children can take small tastes multiple times a day to encourage purposeful swallows and reduce posterior drooling (24). They should also have meals with their family. On the rare circumstance a child has a tracheostomy, laryngotracheal separation can be considered, following which, the individual is unable to speak (36).

### Oropharyngeal Dysphagia: Aspiration of Saliva

Drooling is considered abnormal after age 4. It can be anterior (out of the mouth) or posterior (pooling of saliva around the larynx). Drooling is common in CP. Increased salivation can result from some medications and impairments (including temporal lobe seizures, autonomic, and Rolandic epilepsy, medications like benzodiazepines, especially clonazepam, and irritation factors like teething, and GER). Children with dyskinetic CP have more intense drooling than children with spastic CP. However, children with CP do not generally produce more saliva than children without CP (37). Drooling in CP is therefore a swallowing problem, caused by poor voluntary control of head, lips, tongue, and jaw, decreased sensation, and decreased swallowing frequency.

Anterior drooling occurs in 40% of children with CP (38), with severe drooling in 15%. It is significantly associated with intellectual disability, epilepsy, non-spastic motor type, quadriplegia, or hemiplegia, limited speech, poor lip closure, and feeding difficulties.

Anterior drooling in itself does not cause lung disease, and the treatment goal is usually to improve participation and self-management through behavior modification. However, interventions to decrease anterior drooling may also decrease posterior drooling. There are quantifying systems for anterior, but not posterior drooling. The Drooling Impact Scale is a simple, validated, and quick method for assessing response to treatment (39). Unfortunately, posterior drooling is harder to measure; investigations are similar to those for food aspiration. Most interventions aim to minimize saliva production, so both types of drooling are managed similarly.

Treatments of drooling include conservative, medical and surgical approaches. Conservative management includes physical, oral sensorimotor therapies, behavioral interventions, and intra-oral appliances. These aim to deal with the underlying problem: head and body posture, lip and jaw closure, and tongue control. Behavioral therapies aim to improve self-control of drooling: wiping, head control, swallowing, and mouth closure (40). Intra-oral appliances assist in jaw stability and develop lip and tongue control. If these are not working, there needs to be consultation with the family to decide whether to wait for the skill to be acquired or accept that it will not get better. If medications are required, anticholinergic agents like glycopyrrolate, benzhexol, and hyoscine are commonly used. Side-effects include dry mouth, thick secretions, urinary retention, constipation, facial flushing, and dilated pupils. Most studies show improvement in drooling. Glycopyrrolate has been shown to be superior to hyoscine patches in children with neurodisability (41). However, given the different routes and comorbidities of the child the initial drug chosen may be different (40, 42). Overtreating can lead to unmanageably thick saliva, especially in the morning. No studies have examined the long-term side-effects of these medications.

Botulinum neurotoxin type A (BoNTA) injections into the salivary glands are generally at 4–6 months intervals. Most studies have found a significant reduction in saliva using objective and subjective outcomes (43, 44). Injections are performed under ultrasound guidance into the parotid or submandibular glands or both. Side-effects can be localized from the injection or BoNTA-related. BoNTA-related side-effects include mouth dryness, thick saliva, and worsening of dysphagia (39). Dohar found that BoNTA and either glycopyrrolate or hyoscine combined had better results than either agent alone. No tachyphylaxis occurred (45). A statistically significant reduction in the number of pneumonia episodes post treatment has been reported (45, 46), at least in children without advanced chronic respiratory disease (43).

Surgery to salivary glands may be considered, including submandibular gland excision, duct ligation, or rerouting, sublingual gland excision, parotid duct ligation, and sectioning of the parasympathetic neural pathway. These can be done stepwise to prevent a dry mouth. Submandibular gland rerouting is contraindicated in posterior drooling (47) and sectioning of the parasympathetic neural pathway can cause loss of taste and hearing (40). In some centers, to allow for growth, surgical approaches are not recommended before age 12. In children with anterior drooling and large tonsils and adenoids, removal of these initially may be helpful, especially if associated with sleep disordered breathing. BoNTA and submandibular duct relocation are both effective, but surgery provides a longer, larger effect (48). Uncontrolled studies have shown a reduction in respiratory symptoms after surgery (45, 49–51).

### Gastroesophageal Reflux and Aspiration

Gastroesophageal reflux (GER) is defined as passage of gastric contents into the esophagus with or without regurgitation and vomiting. Gastroesophageal reflux disease (GERD) is defined as GER causing troublesome symptoms and/or complications. The prevalence of GER is quoted as 70–90% (by pH studies and upper gastrointestinal endoscopy) in children with CP and either failure to thrive, food refusal, small volume feeds, or vomiting (35). Causes include slowed gastric emptying, constipation, seizures, esophageal dysmotility, chronic respiratory symptoms, medications, poor posture, and scoliosis, increased intra-abdominal pressure due to abdominal wall spasticity, prolonged supine position, and certain foods (52, 53). Vomiting, rumination and hematemesis are associated with higher risk of GERD in people with severe intellectual disability (54). There are also higher rates of Barrett's esophagus and adenocarcinoma in adults with severe intellectual disability (55).

The European Society for Pediatric Gastroenterology, Hepatology and Nutrition (ESPHAN) guidelines on gastrointestinal and nutritional complications in children with neurological impairment recommend objective measures for diagnosing GERD (56), (e.g., endoscopy with biopsy and esophageal pH studies with or without impedance monitoring). However, given its high prevalence, a trial of a proton pump inhibitor with careful follow-up is also acceptable. The use of extraesophageal biomarkers like lipid laden macrophages and pepsin on bronchoalveolar lavage or bilirubin on esophageal swabs has not been found helpful. Unfortunately, current investigations correlate poorly with symptomatology (57).

Management can include lifestyle modifications, medications, and surgery. Lifestyle advice includes thickened feeds, avoiding caffeine, acidic, fatty or spicy foods, sitting upright during and after eating, or having frequent, small meals, and weight loss if obese (58). Food thickener pectin and whey-based formulas have been found helpful (59, 60). If gastric emptying is delayed, a trial of hydrolysed formula may help. Treating constipation may improve gastric emptying. In terms of medications, proton pump inhibitors (PPI) are generally better than histamine receptor blockers. They both work by reducing acid production, not by preventing reflux. Symptoms should be re-evaluated after 4–8 weeks of treatment. PPI should be weaned before stopping (58). PPI have been linked to respiratory and gastrointestinal infections and bone mineral density loss in adults (61). As PPI do not prevent aspiration, Mertens' study on human bronchial epithelial cells showed increased inflammation from exposure to gastric juice on PPI vs. off PPI (62). Also, Brodlie showed that bile acids can be detected on bronchoalveolar lavage in patients with cystic fibrosis on PPI (63). Therefore, continuous use of PPI maybe detrimental to lung health.

Prokinetics include baclofen, domperidone, metoclopramide, and erythromycin. They help improve gastric emptying and reduce lower esophageal sphincter relaxation. Rosen recommends that baclofen be considered before surgery if other medications have failed (58). Baclofen is commonly used to treat spasticity in children with CP, though at higher doses. There is some evidence that short term use of baclofen reduces the rate of transient lower esophageal sphincter relaxation and increases gastric emptying, hence reducing emesis and reflux in children with and without neurodisability (64, 65). The other prokinetics are not recommended in children owing to side-effects and poor evidence. There is limited evidence that medications for GER improve lung disease. Also, there are interactions between glycopyrrolate and all of the above except erythromycin, making one of the drugs ineffective (66). Medications that delay gastric emptying include antacids, histamine receptor blockers, PPI, ondansetron, anticholinergic medications including antidepressants and oxybutynin, beta blockers, calcium channel blockers, opioids, and levodopa. These should be avoided if possible. As these are commonly prescribed, care must be taken when reviewing patients' medications.

Surgical management includes gastrostomy, jejunostomy, fundoplication, and total esophagogastric disconnection. With current evidence, gastrostomy insertion should occur first. If reflux persists, then medical management should be tried (56, 58). If this fails, gastrojejunostomy tube extension can be tried. Fundoplication and gastrojejunal feeding have similar outcomes (67, 68). Jejunal feeding must be continuous and can be dislodged, requiring radiological intervention for reinsertion. A separate jejunostomy site can be inserted if dislodgement recurs. The data available for conversion from gastrostomy to gastrojejunostomy tube is limited. A study on infants with feeding difficulties showed that only 12% required change to gastrojejunal tube. However, most had underlying cardiac disease (69). When comparing children that ultimately require gastrojejunal feeding, there was no difference in preoperative patient characteristics, hospitalization rates, or diagnostic evaluations at gastrostomy insertion (70). Fundoplication is associated with post-operative complications in up to 59%. Reoccurrence of GER with need for repeat procedures occurs in 5–15%. Other complications include gas bloat syndrome, dumping syndrome, and retching (2, 71). A longitudinal, prospective multicenter cohort study of children with CP admitted in the 6 months prior to gastrostomy insertion reported a reduction in hospitalizations by 26% 6 months following gastrostomy, and a further 7% by 12 months. Findings were similar for children who also had fundoplication (72). A review of hospital admissions pre and post gastrostomy in children with severe intellectual disability found fewer all-cause and epilepsy-related hospitalizations, but not acute lower respiratory tract infections (73).

### Motor Disorder

The disorder of movement and posture is the mainstay of the CP diagnosis. Abnormal movement and posture are due to the upper motor neuron lesion causing positive (e.g., changes in tone) and negative (e.g., weakness) signs (74). Changes in tone include hypertonia (the most common), hypotonia, and hyperkinesis. Both positive and negative signs cause secondary deficits, including contractures, bony and spinal deformities, and poor force production. There are probably multiple causal pathways of respiratory disease from a motor perspective and therefore multiple preventive actions and treatments. This conceptual model provides a contextual approach to therapies and preventative strategies.

Normal respiration depends on coordination of diaphragmatic, abdominal, chest, neck, and throat muscle actions, and structural stability of the trunk and head. This allows forceful inspiration and expiration and an effective cough. A poor cough cannot adequately clear lower airway secretions and protect against aspiration (75, 76). Deep breathing during exercise helps clear secretions and open up atelectasis. Children who cannot exercise vigorously (including many classified as GMFCS V) are prone to atelectasis and infection.

**Figure 2:**
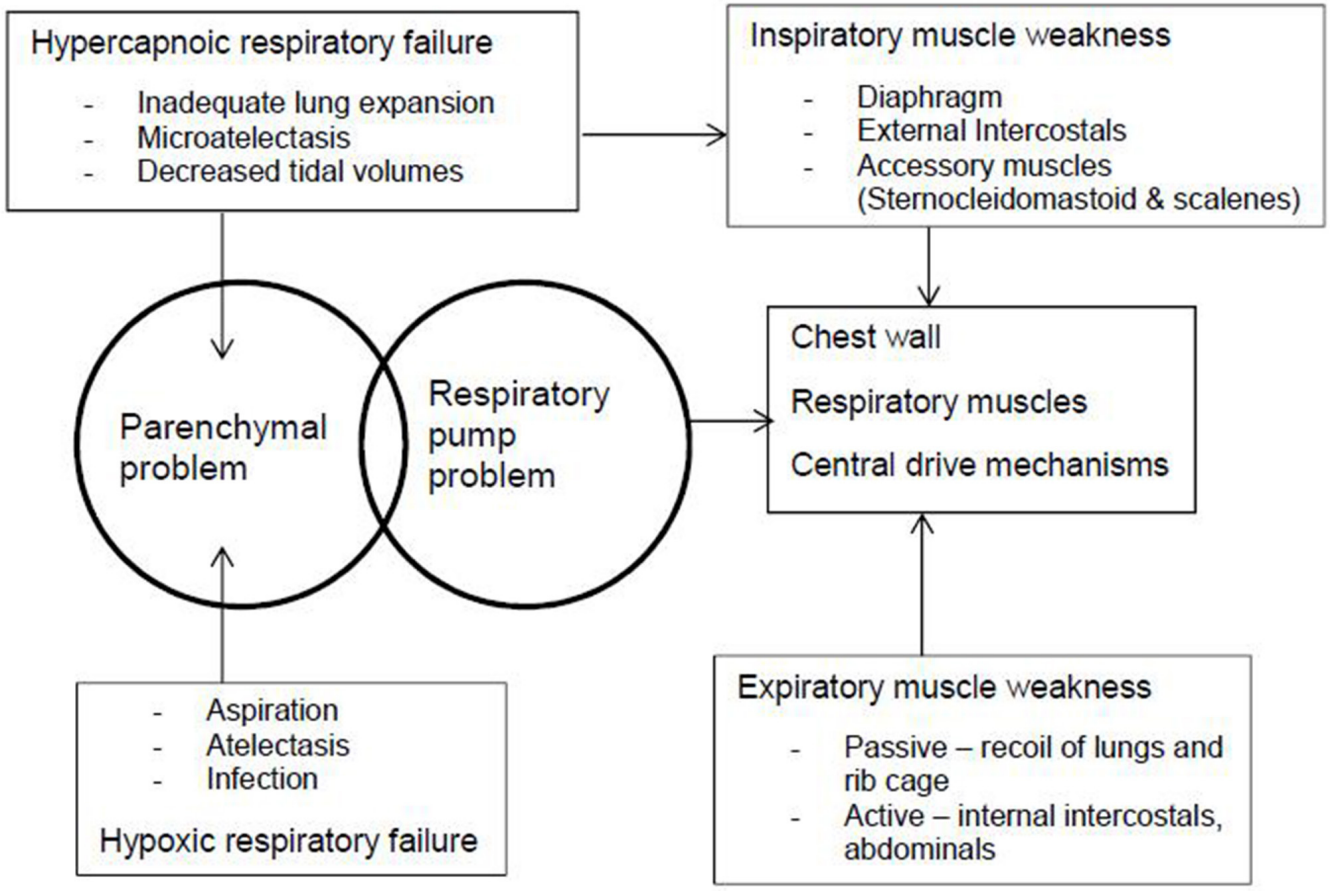
The pathophysiology of respiratory illness in cerebral palsy occurs because of parenchymal and respiratory pump problems. The chest wall, respiratory muscles, and central drive mechanisms cause muscle weakness, which lead to hypercapnoic and hypoxic respiratory failure.

Typical infants learn to hold their head and chest and then roll. Otherwise, chest wall development is impaired (77). Children who cannot sit unsupported cannot co-contract paraspinal and abdominal muscles, resulting in immediate mid-trunk collapse and slumped posture (78), and evolving into fixed kyphoscoliosis over time (Figure 2). Malalignment of the spine from any cause contributes to reduced gas exchange (hypoxemia and hypercapnia), increased resistive load to breathing, and increased risk of upper airway obstruction (UAO), atelectasis, and pneumonia caused by unequal expansion of lungs (29, 79). The increased positive pressures required to counteract this may also cause maladaptive compensatory recruitment of accessory muscles (29). The hypoxemia and vascular bed restriction can cause pulmonary hypertension and cor pulmonale (80). Patients with hypertonia have a weaker diaphragm (81).

GMFCS level predicts risk of respiratory illness. Though GMFCS is still considered non-modifiable, therapy should maximize musculoskeletal function, and minimize any progressive musculoskeletal complications.

Lung function varies, depending on posture. In typically developed adults, expiratory flow is highest when standing, reduced when sitting, and greatly reduced when slumped (82). Children with CP have poorer lung function than their typically developing gender- and height-matched peers (83). Forced expiratory function and vital capacity are worse with increasing GMFCS level (83, 84). Scoliosis also reduces vital capacity in adults with spastic CP (85). Exercise, respiratory feedback, incentive spirometry and resistance exercise improve lung function in CP (86–89). Lung function improves with resistant exercise, swimming and gym, and respiratory feedback (84, 86–89). Maximizing physical activity at all GMFCS levels should be considered (for example, hydrotherapy, standing and walking frames) and may improve lung function. It is unknown whether improving lung function improves respiratory health.

In terms of skeletal malalignment, the main issues are scoliosis, hip displacement and pelvic obliquity. The prevalence of scoliosis in CP is 20–25% (90). Severe scoliosis occurs only in non-ambulant children (74). Lung function is difficult to obtain in children with severe disability, but in adolescent idiopathic scoliosis lung function is worse with large thoracic curves, older age, and thoracic vs. lumbar curves (80). Conservative measures like postural management have not shown benefits in small studies (79, 91). Bracing is not tolerated and BoNTA is ineffective.

Prevention of pelvic obliquity, hip dislocation, and fixed hip deformity may minimize spinal deformity. Current evidence supports long instrumental fusion from T2 to pelvis. If left untreated, scoliosis may progress in adulthood (74). Surgery should be considered when a curve exceeds 50° or functional sitting deteriorates. Surgery corrects deformity and improves child and carer QoL (90). However, motor function or risk of pneumonia do not improve (92). This is probably because motor function and respiratory illness antecede scoliosis. Therefore, whilst there is no evidence that scoliosis causes respiratory illness, it is a marker of weakness and may exacerbate respiratory symptoms by limiting chest expansion, particularly if pain is present.

Hip displacement with pelvic obliquity affects scoliosis development and diaphragmatic movement (74). Hip displacement occurs in one third of children with CP, and 90% of those at GMFCS V. Excessive external hip rotation with posterior pelvic tilt compresses the mid-trunk and pelvic floor, impeding diaphragmatic movement. BoNTA does not prevent hip displacement. Early orthopedic input is important, as it reduces the need for hip salvage surgery (74), which has poor outcomes.

### Airway Clearance

Physiotherapists who specialize in respiratory health can carry out a full subjective and objective assessment, to determine the correct treatment strategy for each individual child with CP. The aims of physiotherapy treatment may include preventing atelectasis, improving lung compliance, maintenance of chest wall mobility and aerobic fitness, airway clearance regimes to aid secretion clearance, and improving cough effectiveness (91, 93). Airway clearance techniques supplement the body's mucociliary clearance system (93, 94).

A basic approach to airway clearance includes unsticking secretions in smaller distal airways, collecting these secretions into larger airways and then evacuating them out of the lungs. Types of treatments include positioning and exercise or active movements where possible, modified autogenic drainage, manual techniques such as percussions and vibrations and positioning and manual techniques to improve cough effectiveness (94). To help loosen sticky secretions, mucolytics nebulisers such as saline or hypertonic saline may be used in conjunction with airway clearance. There are no studies to prove the effectiveness of these physiotherapy treatments in this group of patients (22). Due to the paucity of evidence to assess effectiveness or adverse effects of interventions, it is very important to monitor both the positive and negative outcomes of interventions, particularly ensuring that any techniques that increase secretions, or mobilize secretions from the peripheral to central airways can be safely managed by the patient. Careful observation should be taken of children who are unable to clear secretions by cough. For children with ineffective cough, airway suction, with or without chest physiotherapy, may be required to clear secretions.

High frequency chest wall oscillation and mechanical insufflation-exsufflation may be used to augment chest physiotherapy techniques for airway clearance. High frequency chest wall compression help unstick and collect secretions. Low grade evidence suggests it reduces hospitalizations, improves nocturnal hypoxia and increases need for suctioning (95–98). Families continued it more than conventional chest physiotherapy in a small study (99). There has been one report of a child developing respiratory distress after high frequency chest wall compression (100). As high frequency chest wall compression does not evacuate secretions, it must be applied carefully when patients have an ineffective cough. A device that utilizes a vacuum effect to assist drainage of secretions is reported to accelerate expiratory flow of secretions without creating a negative pressure inside the lungs, therefore preventing airways from collapsing, has been reported to reduce hospital admissions, antibiotics, and visits to primary pediatrician in a small, uncontrolled study (101). Mechanical insufflation-exsufflation can help unstick, collect and evacuate secretions. It shortens the time taken for airway clearance, but is not superior to conventional chest physiotherapy (102). All these studies are low quality and a recent Cochrane review does not recommend changing practice (91).

For children with persistent respiratory symptoms, respiratory physiotherapists educate families/carers in positioning to optimize lung function, maintain chest wall mobility, and if necessary teach home airway clearance regimes. These would include educating parents in manual techniques and ways to improve the effectiveness of cough using, manually assisted cough, cough assist devices, and/or home suction. For children with ineffective cough, airway suction, with or without chest physiotherapy may be required to clear secretions.

### Upper Airway Obstruction and Sleep

Sleep disordered breathing and UAO are common in CP with severe motor impairment (103). Risk factors for sleep disordered breathing include UAO, brainstem dysfunction, epilepsy, chronic aspiration, chest wall deformity, and hypotonia. Treatment of sleep disordered breathing may improve seizure control, reduce lethargy, and improve alertness. However, it increases burden of care, GER, and aspiration risk, and is generally not tolerated. Although widely used, there is no evidence in CP (unlike neuromuscular disease) that non-invasive ventilation prevents pneumonia or hospitalizations. A small study found bilevel positive pressure in acute care reduced length of stay (91).

UAO when awake is potentially life-threatening. It is a multilevel issue involving adenotonsillar hypertrophy, tongue spasticity where reduced tongue mobility causes the tongue to prolapse posteriorly over the larynx, pharyngeal/laryngeal hypotonia, and collapse, and, in some patients, dystonia involving the larynx. Symptoms include noisy and labored breathing, apneas while awake, and neck extension (104). This may lead to multiple and/or prolonged hospitalizations with intensive care admissions. Investigations include a nasoendoscopy to define any surgically correctable factors. Treatment after adenotonsillectomy is difficult, and profound ethical dilemmas exist for families and clinicians. A trial of baclofen may help by reducing spasticity, however too much baclofen can also increase UAO (105). In adults with severe spasticity (not CP related) oral or intrathecal baclofen has not been shown to improve or worsen sleep disordered breathing (106). If no obstructive sleep apnea is present and stridor is absent when asleep, a trial of medication for dystonia or BoNTA could be considered (107). Non-invasive ventilation, adjunct airways like nasopharyngeal airways, and tracheostomy may be appropriate for a few.

### Malnutrition

Malnutrition is common in CP. It can be defined by weight for age, triceps skinfold thickness, mid upper arm fat, arm muscle area, or Dual-energy X-ray absorptiometry (56, 108). Children with severe CP need less energy than their peers. More energy is required in children who were premature with movement disorders and if ambulatory, due to spasticity (56, 109). However, even ambulant children with CP are more sedentary than other children. Obesity should be avoided. Malnutrition causes poor growth, poor general health with more chest infections, more hospitalizations, and carriage of pathogenic bacteria (109–111). The commonest cause of malnutrition is insufficient intake (112), which may be due to miscommunication with the carer, oropharyngeal dysphagia, GERD, constipation, poor positioning with inadequate trunk support, inability to access food independently, increased gag and tonic bite, reduced activity, or medication side-effects resulting in low appetite and constipation. Correcting malnutrition, usually by increased calories or feeding tubes improves constipation, GERD, and overall participation (112, 113). Of note, families sometimes feel their children are less healthy with a feeding tube even when they are nutritionally more healthy (112).

### Carriage of Pathogenic Bacteria

Children with CP appear to carry pathogenic bacteria like Pseudomonas and Klebsiella. However, the data are conflicting. Gerdung's retrospective review showed that 62% of children with CP admitted with pneumonia had gram negative bacteria isolated on respiratory samples and that they tended to do worse (16). Thorburn noted similar findings: in children with CP requiring ventilation for more than 3 days (114), 89% carried Pseudomonas or Klebsiella species. Of these 47% had antibiotic resistances. However, a large prospective study on children with community-acquired pneumonia found that children with neurological conditions were older and more likely to be admitted to intensive care (115). They were less likely to have pathogens detected, and no gram-negative bacteria were isolated in 280 children. A smaller prospective study compared healthy controls and children with neurodisability undergoing elective procedures or admitted to intensive care (116). Bronchoalveolar lavage had higher neutrophil count in more symptomatic children. Streptococcus mitis was commonly identified in the children with neurodisability and no Pseudomonas was identified.

A study of healthy individuals found that oropharyngeal colonization with abnormal bacteria (resolving after 2 weeks) was common during upper respiratory tract infections (117). The Pseudomonas cultured from upper airways in children with CP during a respiratory exacerbation could be similar. Mackowiak reported that pharyngeal colonization of gram-negative bacilli in adults susceptible to aspiration was a risk factor for gram negative bacilli nosocomial respiratory infection (118). Interestingly, adults with epilepsy were not colonized.

A case series on three people with severe neurodisability with recurrent admissions for pneumonia, showed reduced symptoms and no hospitalizations after starting month-on, month-off inhaled tobramycin (97). A double-blinded randomized control trial found no difference in time to discharge between penicillin and clindamycin in children with aspiration pneumonia (119).

In children with bronchiectasis, antibiotic prophylaxis is considered after three exacerbations per year (120). In children with CP with recurrent exacerbations, prophylaxis could also be considered.

### Epilepsy

Uncontrolled seizures may increase salivation, particularly generalized tonic-clonic, autonomic, and Rolandic seizures. Increased salivation and decreased consciousness increase the risk of aspiration. Similarly, mainly due to their side-effect of sedation, antiepileptic drugs also increase risk of aspiration. Benzodiazepines, particularly clonazepam, increase salivation in their own right. Optimal treatment of epilepsy may reduce respiratory illness in CP by reducing aspiration risk.

### Asthma

The prevalence of asthma in people with CP should not be different to that of the general population. A higher prevalence of asthma has been reported in young adults with CP than those without CP (121), possibly because respiratory illness exacerbations are sometimes similar to asthma exacerbations. Moreover, adults with CP are often given a new diagnosis of asthma at the onset of respiratory illness, though it is unknown how often (122). After fundoplication, children with neurological impairment had less hospital admissions for reflux, aspiration pneumonia, GERD, and mechanical ventilation. However, the admissions for pneumonia remained stable and those for asthma increased (123). Therefore, most children with recurrent wheezy respiratory illnesses would benefit from a short trial of asthma treatment. If no benefit is found, this should be discontinued.

## Holistic Care and Lifestyle

There are multiple barriers to providing cohesive evaluation and management of children, including the heterogeneity of the etiologies and severity of CP, the multifactorial components of the risk factors, lack of evidence of interventions, and sometimes the limited cohesion of multidisciplinary teams in providing clear messages and choices for families. The primary aims of management are to improve QoL and function and minimize burden to individuals and their families. The child as a whole person and family need to be considered in all decision-making.

Routine immunizations and annual influenza vaccines should be encouraged, as children with CP are more likely to have incomplete or delayed immunizations than the general population (124).

Children with severe neurological disease use fewer dental services than other children, (125) yet this might be an effective strategy to decrease respiratory illness in children with CP. In a recent study, dental care was the only intervention shown to decrease subsequent admissions for pneumonia in children with neurological impairment (126). This is likely due to reducing carriage of oral flora that is pathogenic to the lower airways. Routine oral hygiene has been seen to reduce ventilator associated pneumonia in intensive care settings and respiratory infections in adults undergoing heart surgery (127, 128).

## Conclusion

Respiratory illness in CP is a common problem leading to high morbidity and mortality in those severely affected. The underlying pathophysiology is still uncertain, probably caused largely by aspiration of food, saliva, and gastric content, with the additional contribution of the motor disorder that gives CP its definition.

We are still struggling with what defines respiratory illness in CP with children with severe CP having multiple admissions for bronchiolitis or asthma. It is commonly thought that aspiration has to be discrete (e.g., choking), not ongoing.

Evidence-based management of respiratory illness is yet to be described. Risk factors are known. They are multifactorial, and interventions directed at these may help. It is likely that a complex care management approach is required due to the multifactorial risks unique to each child. Treatment should be aimed at improving QoL and reducing burden of care in the context of family-based decisions, ethics, and a multidisciplinary team. Treatment needs to be time-limited and regularly reviewed to make sure that it is not causing harm.

## Author Contributions

RM prepared the first draft of this paper, under the supervision of AW. The draft was revised critically by AB, NG, MC, and KL. All authors made substantial contributions to the literature review.

## Conflict of Interest

The authors declare that the research was conducted in the absence of any commercial or financial relationships that could be construed as a potential conflict of interest.

## Abbreviations

BoNTA: Botulinum neurotoxin type A
CP: Cerebral palsy
EDACS: Eating and Drinking Ability Classification System
GER: Gastroesophageal reflux
GERD: Gastroesophageal reflux disease
GMFCS: Gross Motor Function Classification system
QoL: Quality of Life
PPI: Proton pump inhibitors
UAO: Upper airway obstruction
VFSS: Videofluoroscopy swallow study.

## References

[1] Graham HK Selber P Musculoskeletal aspects of cerebral palsy. J Bone Joint Surg Br. (2003) 85–B:157–66. 10.1302/0301-620X.85B2.14066 12678344

[2] Rosenbaum P Paneth N Leviton A Goldstein M Bax M Damiano D. A report: the definition and classification of cerebral palsy April 2006. Dev Med Child Neurol. (2007) 49:8–14. 10.1111/j.1469-8749.2007.tb12610.x 17370477

[3] Australian Cerebral Palsy Register Australian Cerebral Palsy Register Report. (2018). Available online at: https://cpregister.com/wp-content/uploads/2019/02/Report-of-the-Australian-Cerebral-Palsy-Register-Birth-Years-1995-2012.pdf (accessed February 12, 2020).

[4] Himmelmann K Hagberg G Uvebrant P. The changing panorama of cerebral palsy in Sweden. X. Prevalence and origin in the birth-year period 1999-2002. Acta Paediatr Int J Paediatr. (2010) 99:1337–43. 10.1111/j.1651-2227.2010.01819.x 20377538

[5] Palisano R Rosenbaum P Walter S Russell D Wood E GB. Reliability of a system, function in children with cerebral palsy. Dev Med Child Neurol. (1997) 39:214–23. 10.1111/j.1469-8749.1997.tb07414.x 9183258

[6] Blair E Langdon K McIntyre S Lawrence D Watson L. Survival and mortality in cerebral palsy: observations to the sixth decade from a data linkage study of a total population register and national death index. BMC Neurol. (2019) 19:1–11. 10.1186/s12883-019-1343-1 31164086 PMC6549269

[7] Blair E Watson L Badawi N Stanley FJ Life expectancy among people with cerebral palsy in western Australia. Dev Med Child Neurol. (2010) 43:508–15. 10.1111/j.1469-8749.2001.tb00753.x 11508916

[8] Ryan JM Peterson MD Ryan N Smith KJ O'connell NE Liverani S. Mortality due to cardiovascular disease, respiratory disease, and cancer in adults with cerebral palsy. Dev Med Child Neurol. (2019) 61:924–8. 10.1111/dmcn.14176 30727025 PMC6850409

[9] Brooks JC Strauss DJ Shavelle RM Tran LM Rosenbloom L Wu YW. Recent trends in cerebral palsy survival. Part I: Period and cohort effects. Dev Med Child Neurol. (2014) 56:1059–64. 10.1111/dmcn.12520 24966011

[10] Reid SM Carlin JB Reddihough DS. Survival of individuals with cerebral palsy born in victoria, Australia, between 1970 and 2004. Dev Med Child Neurol. (2012) 54:353–60. 10.1111/j.1469-8749.2012.04218.x 22329739

[11] Himmelmann K Sundh V. Survival with cerebral palsy over five decades in western Sweden. Dev Med Child Neurol. (2015) 57:762–7. 10.1111/dmcn.12718 25694102

[12] Meehan E Freed GL Reid SM Williams K Sewell JR Rawicki B Tertiary paediatric hospital admissions in children and young people with cerebral palsy. Child Care Health Dev. (2015) 41:928–37. 10.1111/cch.12263 26032706

[13] Meehan E Reid SM Williams K Freed GL Sewell JR Vidmar S Hospital admissions in children with cerebral palsy: a data linkage study. Dev Med Child Neurol. (2017) 59:512–9. 10.1111/dmcn.13350 27900776

[14] Elema A Zalmstra TAL Boonstra AM Narayanan UG Reinders-Messelink HA Annette AAJ. Pain and hospital admissions are important factors associated with quality of life in nonambulatory children. Acta Paediatr Int J Paediatr. (2016) 105:e419–25. 10.1111/apa.13493 27250697

[15] Vemuri S Baker L Williams K Hynson J. The last 2 years of life for children with severe physical disability: observations from a tertiary paediatric centre. J Paediatr Child Health. (2018) 54:1357–61. 10.1111/jpc.14092 29943874

[16] Gerdung CA Tsang A Yasseen AS Armstrong K Mcmillan HJ Kovesi T. Association between chronic aspiration and chronic airway infection with pseudomonas aeruginosa and other gram-negative bacteria in children with cerebral palsy. Lung. (2016) 194:307–14. 10.1007/s00408-016-9856-5 26883134

[17] Gilman RH Brown KH Gilman J Gaffar A Alamgir S Kibriya A. Colonisation of the oropharynx with gram negative bacilli in children with severe protein calorie malnutrition. Am J Clin Nutr. (1982) 36:284–9. 10.1093/ajcn/36.2.284 6808821

[18] Blackmore AM Bear N Blair E Gibson N Jalla C Langdon K. Prevalence of symptoms associated with respiratory illness in children and young people with cerebral palsy. Dev Med Child Neurol. (2016) 58:780–1. 10.1111/dmcn.13016 27307196

[19] Blackmore AM Bear N Blair E Gibson N Jalla C Langdon K Factors associated with respiratory illness in children and young adults with cerebral palsy. J Pediatr. (2016) 168:151–7.e1. 10.1016/j.jpeds.2015.09.064 26520916

[20] Blackmore AM Langdon K Bear N Wilson AC Steer K Blair E. Predicting respiratory hospital admissions in young people with cerebral palsy. Arch Dis Child. (2018) 103:1119–24. 10.1136/archdischild-2017-314346 29555725 PMC6287554

[21] Blackmore AM Bear N Langdon K Moshovis L Gibson N Wilson A. Respiratory hospital admissions and emergency department visits in young people with cerebral palsy: 5-year follow-up. Arch Dis Child. (2019). 10.1136/archdischild-2019-317714. [Epub ahead of print]. 31256055

[22] Blackmore AM Gibson N Cooper MS Langdon K Moshovis L Wilson AC. Interventions for management of respiratory disease in young people with cerebral palsy: a systematic review. Child Care Health Dev. (2019) 45:1–18. 10.1111/cch.12703 31276598

[23] Myers LL Nerminathan A Fitzgerald DA Chien J Middleton A Waugh MC. Transition to adult care for young people with cerebral palsy. Paediatr Respir Rev. (2020) 33:16–23. 10.1016/j.prrv.2019.12.002 31987717

[24] Arvedson JC Feeding children with cerebral palsy and swallowing difficulties. Eur J Clin Nutr. (2013) 67:S9–12. 10.1038/ejcn.2013.224 24301008

[25] Benfer KA Davies PSW Boyd RN Weir KA Bell KL Ware RS. Oropharyngeal dysphagia and cerebral palsy. Pediatrics. (2017) 140:731. 10.1542/peds.2017-0731 29167377

[26] Benfer KA Weir KA Bell KL Ware RS Davies PS Boyd RN. Oropharyngeal dysphagia and gross motor skills in children with cerebral palsy. Pediatrics. (2013) 131:61–70. 10.1542/peds.2012-3093 23589816

[27] Benfer KA Weir KA Bell KL Ware RS Davies PS Boyd RN. Longitudinal study of oropharyngeal dysphagia in preschool children with cerebral palsy. Arch Phys Med Rehabil. (2016) 97:552–560.e9. 26707458 10.1016/j.apmr.2015.11.016

[28] Benfer KA Weir KA Bell KL Ware RS Davies PSW Boyd RN. Food and fluid texture consumption in a population-based cohort of preschool children with cerebral palsy: relationship to dietary intake. Dev Med Child Neurol. (2015) 57:1056–63. 10.1111/dmcn.12796 25982341

[29] Benfer KA Weir KA Bell KL Ware RS Davies PSW Boyd RN Clincal signs suggestive of pharyngeal dysphagia in preschool children with cerebral palsy. Res Dev Disabil. (2014) 35:3469–81. 10.1016/j.ridd.2014.12.021 25562439

[30] Benfer KA Weir KA Bell KL Ware RS Davies PSW Boyd RN. The eating and drinking ability classification system in a population-based sample of preschool children with cerebral palsy. Dev Med Child Neurol. (2017) 59:647–54. 10.1111/dmcn.13403 28276586

[31] Benfer KA Weir KA Boyd RN. Clinimetrics of measures of oropharyngeal dysphagia for preschool children with cerebral palsy and neurodevelopmental disabilities: a systematic review. Dev Med Child Neurol. (2012) 54:784–9. 10.1111/j.1469-8749.2012.04302.x 22582745

[32] Tanaka N Nohara K Ueda A Katayama T Ushio M Fujii N. Effect of aspiration on the lungs in children: a comparison using chest computed tomography findings. BMC Pediatr. (2019) 19:1–7. 10.1186/s12887-019-1531-6 31117982 PMC6529997

[33] Adams MS. Feeding difficulties in children with cerebral: low cost caregiver training in Dhaka Bangladesh. Child Care Health Dev. (2011) 38:878–88. 10.1111/j.1365-2214.2011.01327.x 22082112

[34] Somerville H Tzannes G Wood J Shun A Hill C Arrowsmith F. Gastrointestinal and nutritional problems in severe developmental disability. Dev Med Child Neurol. (2008) 50:712–6. 10.1111/j.1469-8749.2008.03057.x 18754923

[35] Rogers B. Feeding method and health outcomes of children with cerebral palsy. J Pediatr. (2004) 145:S28–32. 10.1016/j.jpeds.2004.05.019 15292884

[36] Teramoto S. Moral and depression in patients treated surgically fro intractable aspiration. Chest. (2000) 118:564–5. 10.1378/chest.118.2.564-a 10936162

[37] Erasmus CE Van Hulst K Rotteveel LJC Jongerius PH Van Den Hoogen FJA Roeleveld N Drooling in cerebral palsy: hypersalivation or dysfunctional oral motor control? Dev Med Child Neurol. (2009) 51:454–9. 10.1111/j.1469-8749.2008.03243.x 19207297

[38] Reid SM Mccutcheon J Reddihough DS Johnson H. Prevalence and predictors of drooling in 7- to 14-year-old children with cerebral palsy : a population study. Dev Med Child Neurol. (2012) 54:1032–6. 10.1111/j.1469-8749.2012.04382.x 22881219

[39] Reddihough D Erasmus CE Johnson H McKellar GMW Jongerius PH. Botulinum toxin assessment, intervention and aftercare for paediatric and adult drooling: international consensus statement. Eur J Neurol. (2010) 17:109–21. 10.1111/j.1468-1331.2010.03131.x 20633182

[40] Walshe M Smith M Pennington L. Interventions for drooling in children with cerebral palsy (Review). Cochrane Collab. (2012) 11:CD008624. 10.1002/14651858.CD008624.pub2 23152263 PMC11664232

[41] Parr JR Todhunter E Pennington L Stocken D Cadwgan J O'Hare AE. Drooling Reduction Intervention randomised trial (DRI): comparing the efficacy and acceptability of hyoscine patches and glycopyrronium liquid on drooling in children with neurodisability. Arch Dis Child. (2018) 103:371–6. 10.1136/archdischild-2017-313763 29192000 PMC5890631

[42] Bachrach S Walter R. Use of glycopyrrolate and other anticholinergic medications for. Clin Pediatr. (1998) 37:485–90. 10.1177/000992289803700805 9729704

[43] Gubbay A Marie Blackmore A. Effects of salivary gland botulinum toxin-A on drooling and respiratory morbidity in children with neurological dysfunction. Int J Pediatr Otorhinolaryngol. (2019) 124:124–8. 10.1016/j.ijporl.2019.05.044 31185343

[44] Meece R Fishlock K Bayley E Keller M Ultrasound-Guided botox injections of salivary glands in children with drooling. J Radiol Nurs. (2010) 29:20–4. 10.1016/j.jradnu.2009.12.002

[45] Dohar JE. Sialorrhea & aspiration control - a minimally invasive strategy uncomplicated by anticholinergic drug tolerance or tachyphylaxis. Int J Pediatr Otorhinolaryngol. (2019) 116:97–101. 10.1016/j.ijporl.2018.10.035 30554718

[46] Faria J Harb J Hilton A Yacobucci D Pizzuto M. Salivary botulinum toxin injection may reduce aspiration pneumonia in neurologically impaired children. Int J Pediatr Otorhinolaryngol. (2018) 79:2124–8. 10.1016/j.ijporl.2015.09.029 26478107

[47] Sousa S Salgado C Santos M dos Reis S Pereira G Patrão F. Submandibular duct transposition for drooling in children: a Casuistic review and evaluation of grade of satisfaction. Int J Pediatr Otorhinolaryngol. (2018) 113:58–61. 10.1016/j.ijporl.2018.07.023 30174011

[48] Scheffer ART Erasmus C Van Hulst K Van Limbeek J Rotteveel JJ Jongerius PH. Botulinum toxin versus submandibular duct relocation for severe drooling. Dev Med Child Neurol. (2010) 52:1038–42. 10.1111/j.1469-8749.2010.03713.x 20561006

[49] Khadivi E Bakhshaee M Ashraf zadeh F Afzal Aghaee M Movahed SR Nabavi SS. Bilateral submandibular duct rerouting: assessment of results on drooling in cerebral palsy cases. Auris Nasus Larynx. (2013) 40:487–90. 10.1016/j.anl.2013.01.007 23489831

[50] Manrique D Sato J. Salivary gland surgery for control of chronic pulmonary aspiration in children with cerebral palsy. Int J Lang Commun Disord. (2009) 73:1192–4. 10.1016/j.ijporl.2009.05.002 19535155

[51] Pena AH Cahill AM Gonzalez L Baskin KM Kim H Towbin RB. Botulinum toxin a injection of salivary glands in children with drooling and chronic aspiration. J Vasc Interv Radiol. (2009) 20:368–73. 10.1016/j.jvir.2008.11.011 19157908

[52] Morton RE Wheatley R Minford J. Respiratory tract infections due to direct and reflux aspiration in children with severe neurodisability. Dev Med Child Neurol. (1999) 41:329–34. 10.1017/S0012162299000729 10378759

[53] Srivastava R Jackson WD Barnhart DC. Dysphagia and gastroesophageal reflux disease: dilemmas in diagnosis and management in children with neurological impairment. Pediatr Ann. (2010) 39:225–31. 10.3928/00904481-20100318-07 20411900

[54] de Veer AJE Bos JT Boer R Böhmer CJM Francke AL. Symptoms of gastroesophageal reflux disease in severely mentally retarded people: a systematic review. BMC Gastroenterol. (2008) 8:23. 10.1186/1471-230X-8-23 18547405 PMC2435531

[55] Van Blankenstein M Böhmer CJM Hop WCJ. The incidence of adenocarcinoma in barrett's esophagus in an institutionalized population. Eur J Gastroenterol Hepatol. (2004) 16:903–9. 10.1097/00042737-200409000-00015 15316416

[56] Romano C Van Wynckel M Hulst J Broekaert I Bronsky J Dall'Oglio L. European society for paediatric gastroenterology, hepatology and nutrition guidelines for the evaluation and treatment of gastrointestinal and nutritional complications in children with neurological impairment. J Pediatr Gastroenterol Nutr. (2017) 65:242–64. 10.1097/MPG.0000000000001646 28737572

[57] Tighe M Afzal NA Bevan A Hayen A Munro A Beattie MR Pharmacological treatment of children with gastro-oesophageal reflux. (2014) CD008550. 10.1002/14651858.CD008550.pub2 PMC8947620 25419906

[58] Rosen R Vandenplas Y Singendonk M Cabana M Dilorenzo C Gottrand F Pediatric gastroesophageal reflux clinical practice guidelines: joint recommendations of the north american society for pediatric gastroenterology, hepatology, and nutrition and the european society for pediatric gastroenterology, hepatology, and nutritio. J Pediatr Gastroenterol Nutr. (2018) 66:516–54. 10.1097/MPG.0000000000001889 29470322 PMC5958910

[59] Khoshoo V Zembo M King A Dhar M Reifen R Pencharz P. Incidence of gastroesophageal reflux with whey- and casein-based formulas in infants and in children with severe neurological impairment. J Pediatr Gastroenterol Nutr. (1996) 22:48–55. 10.1097/00005176-199601000-00008 8788287

[60] Miyazawa R Tomomasa T Kaneko H Arakawa H Shimizu N Morikawa A. Effects of pectin liquid on gastroeosphageal reflux disease in children with cerebral palsy. BMC Gastroenterol. (2008) 8:11. 10.1186/1471-230X-8-11 18412980 PMC2383913

[61] Chung EY Yardley J. Are there risks associated with empiric acid suppression treatment of infants and children suspected of having gastroesophageal reflux disease? Hosp Pediatr. (2013) 3:16–23. 10.1542/hpeds.2012-0077 24319831

[62] Mertens V Blondeau K Pauwels A Vos R Vanaudenaerde BM Van Raemdonck DE. 688 effect of gastric juice from patients “on” acid suppressive therapy (PPI) on human bronchial epithelial cells. Gastroenterology. (2009). 136:A−107. 10.1016/S0016-5085(09)60483-2 20216077

[63] Brodlie M Aseeri A Lordan JL Robertson AGN McKean MC Corris PA. Bile acid aspiration in people with cystic fibrosis before and after lung transplantation. Eur Respir J. (2015) 46:1820–3. 10.1183/13993003.00891-2015 26493787 PMC4664606

[64] Omari T Benninga M Sansom L Butler R Dent J Davidson G. Effect of baclofen on eosphagogastric motility and gastroesophageal reflux in children with gastroesophageal reflux disease: a randomised controlled trial. J Pediatr. (2006) 149:468–74. 10.1016/j.jpeds.2006.05.029 17011315

[65] Kawai M Kawahara H Hirayama S Yoshimura N Ida S. Effect of baclofen on emesis and 24-h esophageal ph in neurologically impaired children with gastroesophageal reflux disease. J Pediatr Gastroenterol Nutr. (2004) 38:317–23. 10.1097/00005176-200403000-00017 15076634

[66] Australia M Glycopyrrolate. MIMS. (2020). Available online at: https://www.mimsonline.com.au

[67] Srivastava R Downey EC O'Gorman M Feola P Samore M Holubkov R Impact of fundoplication versus gastrojejunal feeding tubes on mortality and in preventing aspiration pneumonia in young children with neurologic impairment who have gastroesophageal reflux disease. Pediatrics. (2009) 123:338–45. 10.1542/peds.2007-1740 19117901

[68] Stone B Hester G Jackson D Richardson T Hall M Gouripeddi R Effectiveness of fundoplication or gastrojejunal feeding in children with neurologic impairment. Hosp Pediatr. (2017) 7:140–8. 10.1542/hpeds.2016-0126 28159744

[69] Yu YR Cunningham ME DeMello AS Chiou EH Kougias P Wesson DE. Cost-Effectiveness analysis of the surgical management of infants less than one year of age with feeding difficulties. J Pediatr Surg. (2020) 55:187–93. 10.1016/j.jpedsurg.2019.09.076 31759653

[70] McSweeney ME Kerr J Amirault J Fishman E Lurie M Peinado-Fabregat MI Preoperative evaluation is not predictive of transpyloric feeding conversion in gastrostomy-dependent pediatric patients. J Pediatr Gastroenterol Nutr. (2018) 66:887–92. 10.1097/MPG.0000000000001866 29261527 PMC5963971

[71] Sullivan P. Gastrostomy feeding in the disabled child: when is an antireflux procedure required? Arch Dis Child. (1999) 81:463–4. 10.1136/adc.81.6.463 10569957 PMC1718145

[72] Sullivan PB Juszczak E Bachlet AME Lambert B Vernon-Roberts A Grant HW. Gastrostomy tube feeding in children with cerebral palsy: A prospective, longitudinal study. Dev Med Child Neurol. (2005) 47:77–85. 10.1017/S0012162205000162 15707230

[73] Jacoby P Wong K Srasuebkul P Glasson EJ Forbes D Ravikumara M. Risk of hospitalizations following gastrostomy in children with intellectual disability. J Pediatr. (2020) 217:131–8.e10. 10.1016/j.jpeds.2019.10.020 31812294

[74] Kerr Graham H Rosenbaum P Paneth N Dan B Lin J-P Damiano D Cerebral palsy. In Handb Clin Neurol. (2018) 2:15082 10.1038/nrdp.2015.82 PMC9619297 27188686

[75] Proesmans M Vreys M Huenaerts E Haest E Coremans S Vermeulen F. Respiratory morbidity in children with profound intellectual and multiple disability. Pediatr Pulmonol. (2015) 50:1033–8. 10.1002/ppul.23114 25327770

[76] Seddon PC Khan Y. Respiratory problems in children with neurological impairment. Arch Dis Child. (2002) 88:75–8. 10.1136/adc.88.1.75 12495971 PMC1719284

[77] Massery M Multisystem consequences of impaired breathing mechanics and/or postural control. Cardiovasc Pulmonary Phys Ther Evid Practice. (2006) 20:695–718. 10.1097/01823246-200920020-00004

[78] Barks L. Therapeutic positioning, wheelchair seating and pulmonary function of children with cerebral palsy: a research synthesis. Rehabil Nurs. (2004) 29:146–53. 10.1002/j.2048-7940.2004.tb00337.x 15468739

[79] Barks L Davenport P. Wheelchair components and pulmonary function in children with cerebral palsy. Assist Technol. (2012) 24:78–86. 10.1080/10400435.2012.659793 22876730

[80] Johari J Sharifudin MA Ab Rahman A Omar AS Abdullah AT Nor S. Relationship between pulmonary function and degree of spinal deformity, location of apical vertebrae and age among adolescent idiopathic scoliosis patients. Singapore Med J. (2016) 57:33–8. 10.11622/smedj.2016009 26831315 PMC4728701

[81] Bennett S Siritaratiwat W Tanrangka N Bennett MJ Kanpittaya J. Diaphragmatic mobility in children with spastic cerebral palsy and differing motor performance levels. Respir Physiol Neurobiol. (2019) 266:163–70. 10.1016/j.resp.2019.05.010 31125702

[82] Lin F Parthasarathy S Taylor SJ Pucci D Hendrix RW Makhsous M. Effect of different sitting postures on lung capacity, expiratory flow, and lumbar lordosis. Arch Phys Med Rehabil. (2006) 87:504–9. 10.1016/j.apmr.2005.11.031 16571389

[83] Kwon YH Lee HY. Differences of respiratory function in children with spastic diplegic and hemiplegic cerebral palsy, compared with normally developed children. J Pediatr Rehabil Med. (2013) 6:113–7. 10.3233/PRM-130246 23803344

[84] Hutzler Y Chacham A Bergman U. Effects of a movement and swimming program on vital capacity and water orientation skills of children with cerebral palsy. Dev Med Child Neurol. (1998) 40:176–81. 10.1111/j.1469-8749.1998.tb15443.x 9566654

[85] Lampe R Blumenstein T Turova V Alves-Pinto A. Lung vital capacity and oxygen saturation in adults with cerebral palsy. Patient Prefer Adherence. (2014) 8:1691–7. 10.2147/PPA.S72575 25525345 PMC4267512

[86] Kwon H-Y Kim B-J. Effects of task-specific movement patterns during resistance exercise on the respiratory functions and thickness of abdominal muscles of children with cerebral palsy: randomized placebo-controlled double-blinded clinical trial. J Phys Ther Sci. (2018) 30:1073–80. 10.1589/jpts.30.1073 30154603 PMC6110216

[87] Choi JY Rha D Park ES. Change in pulmonary function after incentive spirometer exercise in children with spastic cerebral palsy : a randomized controlled study. Yonsei Med J. (2016) 57:769–75. 10.3349/ymj.2016.57.3.769 26996580 PMC4800370

[88] Lee HY Cha YJ Kim K. The effect of feedback respiratory training on pulmonary function of children with cerebral palsy: a randomised controlled preliminary report. Clin Rehabil. (2014) 28:965–71. 10.1177/0269215513494876 23897949

[89] Lee HY Kim K. Can walking ability enhance the effectiveness of breathing exercise in children with spastic cerebral palsy? J Phys Ther Sci. (2014) 26:539–42. 10.1589/jpts.26.539 24764629 PMC3996417

[90] Toovey R Harvey A Johnson M Baker L Williams K. Outcomes after scoliosis surgery for children with cerebral palsy: a systematic review. Dev Med Child Neurol. (2017) 59:690–8. 10.1111/dmcn.13412 28262923

[91] Winfield NR Barker NJ Quin GL Turner ER. Non-pharmaceutical management of respiratory morbidity in children with severe global developmental delay. Cochrane Database Syst Rev. (2013) 2014:CD010382. 10.1002/14651858.CD010382 25326792 PMC6435315

[92] Keskinen H Lukkarinen H Korhonen K Jalanko T Koivusalo A Helenius I. The lifetime risk of pneumonia in patients with neuromuscular scoliosis at a mean age of 21 years: the role of spinal deformity surgery. J Child Orthop. (2015) 9:357–64. 10.1007/s11832-015-0682-8 26350797 PMC4619373

[93] Schechter MS. Airway clearance applications in infants and children. Respir Care. (2007) 52:1382–91. 17894905

[94] McIlwaine M Bradley J Elborn JS Moran F. Personalising airway clearance in chronic lung disease. Eur Respir Rev. (2017) 26:160086. 10.1183/16000617.0086-2016 28223396 PMC9488523

[95] Fitzgerald K Dugre J Pagala S Homel P Marcus M Kazachkov M. High frequence chest wall compression therapy in neurological impaired children. Respir Care. (2014) 59:107–12. 10.4187/respcare.02446 23777653

[96] Maher A Biney-Amissah F Cycak S Yalamanchi K Defeo J Strategies to reduce the incidence of pneumonia in a pediatric long term care population. AJIC. (2006) 19:E147 10.1016/j.ajic.2006.05.028

[97] Plioplys A V Kasnicka I. Nebulized tobramycin: Prevention of pneumonias in patients with severe cerebral palsy. J Pediatr Rehabil Med. (2011) 4:155–8. 10.3233/PRM-2011-0168 21955974

[98] Javanbakht M Mashayekhi A Montazeri M Rezai Hemami M Branagan-Harris M The vest^TM^ high-frequency chest wall oscillation system compared with manual chest wall physiotherapy for managing airway clearance in patients with complex neurological disorders: a UK-based cost-effectiveness analysis. Open Pharmacoeconomics Heal Econ J. (2019) 7:1–8. 10.2174/1874129001907010001

[99] Yuan N Kane P Shelton K Matel J Becker B Moss RB. Safety, tolerability and efficacy of high frequency chest wall oscillation in pediatric patients with cerebral palsy and neuromuscular diseases: an exploratory randomised controlled trial. J Child Neurol. (2010) 25:815–21. 10.1177/0883073809350223 20357238

[100] Willis LD Warren RH. Acute hypoxaemia in a child with neurologic impairment assocaised with high frequency chest wall compression. Respir Care. (2007) 52:1027–9. 17650359

[101] Garuti G Verucchi E Fanelli I Giovannini M Winck JC Lusuardi M. Management of bronchial secretions with free aspire in children with cerebral palsy : impact on clinical outcomes and healthcare resources. Ital J Pediatr. (2016) 42:7. 10.1186/s13052-016-0216-0 26791415 PMC4719384

[102] Siriwat R Deerojanawong J Sritippayawan S Hantragool S Cheanprapai P. Mechanical insufflation-Exsufflation versus conventional chest physiotherapy in children with cerebral palsy. Respir Care. (2018) 63:187–93. 10.4187/respcare.05663 29066586

[103] Grychtol R Chan EY. Use of non-invasive ventilation in cerebral palsy. ARCH Dis Child. (2018) 103:1170–7. 10.1136/archdischild-2017-313959 29886412

[104] Wilkinson DJ Baikie G Berkowitz RG Reddihough DS. Awake upper airway obstruction in children with spastic quadriplegic cerebral palsy. J Paediatr Child Health. (2006) 42:44–8. 10.1111/j.1440-1754.2006.00787.x 16487389

[105] Revol B Jullian-Desayes I Bailly S Mallaret M Tamisier R Agier MS. Baclofen and sleep apnoea syndrome: analysis of vigibase, the WHO pharmacovigilance database. Eur Respir J. (2018) 51:10–2. 10.1183/13993003.01855-2017 29326335

[106] Bensmail D Salva MAQ Roche N Benyahia S Bohic M Denys P. Effect of intrathecal baclofen on sleep and respiratory function in patients with spasticity. Neurology. (2006) 67:1432–6. 10.1212/01.wnl.0000239827.38036.23 17060570

[107] Worley G Witsell DL Hulka G. Laryngeal dystonia causing inspiratory stridor in children with cerebral palsy. Laryngoscope. (2003) 113:2192–5. 10.1097/00005537-200312000-00028 14660926

[108] Bell KL Benfer KA Ware RS Patrao TA Garvey JJ Arvedson JC. Development and validation of a screening tool for feeding/swallowing difficulties and undernutrition in children with cerebral palsy. Dev Med Child Neurol. (2019) 61:1175–81. 10.1111/dmcn.14220 30937885 PMC6850582

[109] Campanozzi A Capano G Miele E Romano A Scuccimarra G Del Giudice E. Impact of malnutrition on gastrointestinal disorders and gross motor abilities in children with cerebral palsy. Brain Dev. (2007) 29:25–9. 10.1016/j.braindev.2006.05.008 16843628

[110] Huysentruyt K Geeraert F Allemon H Prinzie P Roelants M Ortibus E. Nutritional red flags in children with cerebral palsy. Clin Nutr. (2019) 39:548–53. 10.1016/j.clnu.2019.02.040 30902487

[111] Walson JL Berkley JA. The impact of malnutrition on childhood infections. Curr Opin Infect Dis. (2018) 31:231–6. 10.1097/QCO.0000000000000448 29570495 PMC6037284

[112] Fung EB Samson-Fang L Stallings VA Conaway M Liptak GS Henderson RC. Feeding dysfunction is associated with poor growth and health status in children wit. J Am Diet Assoc. (2002) 102:361–8. 10.1016/S0002-8223(02)90084-2 11902369

[113] Day SM Strauss DJ Vachon PJ Rosenbloom L Shavelle RM Wu YW. Growth patterns in a population of children and adolescents with cerebral palsy. Dev Med Child Neurol. (2007) 49:167–71. 10.1111/j.1469-8749.2007.00167.x 17355471

[114] Thorburn K Jardine M Taylor N Reilly N Sarginson RE Van Saene HKF. Antibiotic-resistant bacteria and infection in children with cerebral palsy requiring mechanical ventilation. Pediatr Crit Care Med. (2009) 10:222–6. 10.1097/PCC.0b013e31819368ac 19057452

[115] Millman AJ Finelli L Bramley AM Peacock G Williams DJ Arnold SR. Community-acquired pneumonia hospitalization among children with neurologic disorders. J Pediatr. (2016) 173:188–95.e4. 10.1016/j.jpeds.2016.02.049 27017483 PMC4897771

[116] Trinick RE Bunni L Thorburn K Hackett AP Dalzell M McNamara PS. An observational study examining the relationship between respiratory symptoms, airway inflammation and bacteriology in children with severe neurodisability. PLoS ONE. (2015) 10:1–12. 10.1371/journal.pone.0124627 25853250 PMC4390362

[117] Ramirez-Ronda C Fuxench-Lopez Z Nevarez M. Increased pharyngeal bacterial colonisation during viral illness. Arch Interna Med. (1981) 141:1599–603. 10.1001/archinte.1981.00340130043013 7305570

[118] Mackowiak P Martin RM Jones SR Smith J. Pharyngeal colonisation by gram negative bacilli in aspiration prone persons. Arch Interna Med. (1978) 138:1224–7. 10.1001/archinte.1978.03630330024009 677978

[119] Jacobson SJ Griffiths K Diamond S Winders P Sgro M Feldman W. A randomized controlled trail of penicillin vs clindamycin for the treatment of aspiration pneumonia in children. (2017) 151:701–4. 10.1001/archpedi.1997.02170440063011 9232045

[120] Chang AB Bush A Grimwood K Bronchiectasis in children: diagnosis and treatment. Lancet. (2018) 392:866–79. 10.1016/S0140-6736(18)31554-X 30215382

[121] Whitney DG Hurvitz EA Ryan JM Devlin MJ Caird MS French ZP. Noncommunicable disease and multimorbidity in young adults with cerebral palsy. Clin Epidemiol. (2018) 10:511–9. 10.2147/CLEP.S159405 29750055 PMC5935087

[122] Fortuna RJ Holub A Turk MA Meccarello J Davidson PW. Health conditions, functional status and health care utilization in adults with cerebral palsy. Fam Pract. (2018) 35:661–70. 10.1093/fampra/cmy027 29718268

[123] Srivastava R Berry JG Hall M Downey EC O'Gorman M Dean JM. Reflux related hospital admissions after fundoplication in children with neurological impairment: retrospective cohort study. BMJ. (2010) 340:85. 10.1136/bmj.b4411 19923145 PMC2779335

[124] Greenwood VJ Crawford NW Walstab JE Reddihough DS. Immunisation coverage in children with cerebral palsy compared with the general population. J Paediatr Child Health. (2013) 49:137–41. 10.1111/jpc.12097 23360148

[125] Chi DL Raklios NA. The relationship between body system-based chronic conditions and dental utilization for medicaid-enrolled children: a retrospective cohort study. BMC Oral Health. (2012) 12:28. 10.1186/1472-6831-12-28 22870882 PMC3433353

[126] Lin JL Van Haren K Rigdon J Saynina O Song H Buu MMC. Pneumonia prevention strategies for children with neurologic impairment. Pediatrics. (2019) 144:e20190543. 10.1542/peds.2019-0543 31537634 PMC9595175

[127] Hoshijima H Kuratani N Takeuchi R Shiga T Masaki E Doi K Effects of oral hygiene using chlorhexidine on preventing ventilator-associated pneumonia in critical-care settings: A meta-analysis of randomized controlled trials. J Dent Sci. (2013) 8:348–57. 10.1016/j.jds.2012.11.004

[128] DeRiso AJ Ladowski JS Dillon TA Justice JW Peterson AC. Chlorhexidine gluconate 0.12% oral rinse reduces the incidence of total nosocomial respiratory infection and nonprophylactic systemic antibiotic use in patients undergoing heart surgery. Chest. (1996) 109:1556–61. 10.1378/chest.109.6.1556 8769511

